# A Putative Novel Hepatitis E Virus Genotype 3 Subtype Identified in Rabbit, Germany 2016

**DOI:** 10.3390/v13061065

**Published:** 2021-06-03

**Authors:** Filip Cierniak, Felicitas von Arnim, Gerald Heckel, Rainer G. Ulrich, Martin H. Groschup, Martin Eiden

**Affiliations:** 1 Institute of Novel and Emerging Infectious Diseases, Friedrich-Loeffler-Institut, 17493 Greifswald-Insel Riems, Germany; filip.cierniak@outlook.de (F.C.); felicitas.var@mail.de (F.v.A.); rainer.ulrich@fli.de (R.G.U.); martin.groschup@fli.de (M.H.G.); 2 Institute of Ecology and Evolution, University of Bern, 3012 Bern, Switzerland; gerald.heckel@iee.unibe.ch; 3 Partner Site Hamburg-Lübeck-Borstel-Riems, Deutsches Zentrum für Infektionsforschung (DZIF), 17493 Greifswald-Insel Riems, Germany

**Keywords:** hepatitis E virus, novel genotype, rabbit

## Abstract

Hepatitis E is an emerging viral disease that is the leading cause of viral hepatitis in the world. The vast majority of hepatitis E cases in developed countries are caused by zoonotic genotypes 3 and 4 of hepatitis E virus (HEV) for which pig and wild boar and to lesser extent rabbits are the main reservoir. According to recent reports rabbits are a source of human HEV infection and highlight the risk of zoonotic foodborne transmission. Here we report the molecular analysis of a novel HEV strain identified in a rabbit during a countrywide surveillance of rabbits and hares in Germany, 2016. The analysis of the complete genome reveals characteristics of a putative novel recombinant subtype of the species *Orthohepevirus A* within the clade of genotype 3 but not closely related to any known subtypes. Importantly, the genome of this strain possesses a nucleotide exchange in the overlapping region of open reading frames ORF2/ORF3 interfering with a broadly applied diagnostic real-time RT-PCR. In conclusion, a new type of HEV strain was identified in a German rabbit with atypical and novel sequence characteristics.

## 1. Introduction

Hepatitis E virus (HEV) is the causative agent of hepatitis E, leading to waterborne epidemics in resource-poor countries and sporadic cases in industrialized countries [1]. HEV belongs to the family *Hepeviridae*, genus *Orthohepevirus*. HEV is a small virus with an RNA genome of positive polarity. The genome of approximately 7500 nucleotides contains three open reading frames (ORF), untranslated regions (UTR) at the 5′ and 3′ ends, and a polyA tract at the 3′ end. ORF1 encodes a nonstructural polyprotein, ORF2 encodes the capsid protein, and ORF3 encodes a small accessory protein, which acts as viroporin [2]. HEV particles were described as naked, non-enveloped virions that are shed into feces as well as membrane-associated quasi-enveloped forms that circulate in blood [3]. Typically, the course of disease is self-limiting and subclinical [4]. Due to the large number of infections, however, HEV remains a constant public health threat with an estimated 20 million cases of hepatitis E, including 3.4 million symptomatic cases and 70,000 deaths per year [5].

Hepeviruses are grouped within four *Orthohepevirus* species that were detected in birds (species *Orthohepevirus B*), rodents and carnivores (species *Orthohepevirus C*), bats (species *Orthohepevirus D*), and genus *Piscihepeviru*s with a fish-associated strain [6]. The species *Orthohepevirus A* is further divided into eight major genotypes and subsequent subgenotypes [7] and is mainly found in humans, domestic pigs, wild boar, deer, and rabbits.

Genotypes 1 and 2 (HEV-1 and HEV-2) occur in Africa and Asia, are exclusively associated with humans, and are transmitted via the fecal–oral route mainly through consumption of contaminated water [8]. In contrast, autochthonous hepatitis E in developed countries is mainly caused by the zoonotic genotypes 3 (HEV-3) and 4 (HEV-4) [1]. HEV-3 strains typically trigger mild disease with rare cases of fulminant hepatitis. However, in immunocompromised individuals or patients with pre-existing conditions the infection can lead to prolonged or chronic forms and final liver failure [4]. Recent estimates for Europe show prevalences between 4.6% and 29.5% depending on the country surveyed (as reviewed by [9]). Pigs and wild boar are the main reservoirs for zoonotic HEV and transmission is generally assumed to occur by ingestion of food products from infected animals [10]. Rabbit HEV was first detected in farmed rex rabbits in China in 2009 [11], and subsequently in farmed and wild rabbits from several other countries, including USA, Germany, the Netherlands, and France [12,13,14,15]. It forms a distinct phylogenetic group within genotype HEV-3, designated HEV-3ra [7] and harbors a characteristic 90/93 nucleotide (nt) insertion within the coding region of ORF1 [14]. There is evidence for zoonotic potential due to reported rabbit HEV infections in humans, most likely affecting immunocompromised patients. Interestingly, direct contact with rabbits does not appear to be necessary for infection with rabbit HEV [16].

During surveillance of hare and rabbits throughout Germany in 2016, several rabbit-derived HEV sequences were recovered [12]. A partial HEV sequence of one individual rabbit indicated the presence of an atypical strain (rab81) outside the rabbit-associated subgenotype HEV-3ra. In this study, we determined the full-length genome of this strain followed by a comprehensive phylogenetic analysis and demonstrated that it represents a unique novel strain within the genotype HEV-3.

## 2. Materials and Methods

### 2.1. RNA Isolation

Frozen liver samples were thawed, and subsequently a small fragment (approximately 30 mg) was transferred to a 1.5 mL tube. Initially, the samples were homogenized in RLT buffer using a TissueLyser II (Qiagen, Hilden, Germany) and RNA extraction was performed with the Qiagen RNeasy Kit according to the manufacturer’s instructions. Alternatively, the samples were homogenized in 750 µL of TriZol LS reagent followed by addition of chloroform and RNA extraction from the aqueous phase. The aqueous phase was mixed with an equal volume of 75% ethanol and loaded on a RNeasy column. Subsequent washing and elution were done according to the RNeasy Kit protocol.

### 2.2. Reverse Transcription-Polymerase Chain Reaction (RT-PCR)

Initially, we used the SuperScript III One-Step RT-PCR Kit (ThermoFisher, Hennigsdorf, Germany) for reverse transcription (RT) with target-specific oligonucleotides and PCR according to the manufacturer. Alternatively, the SuperScript IV First-Strand Synthesis Kit (ThermoFisher) was used for RT with random hexamers followed by polymerase chain reaction (PCR) using Phusion DNA polymerase (ThermoFisher) with HEV-specific primers. PCR products were purified using the Qiagen PCR Purification Kit or were extracted after agarose gel electrophoresis using the Qiagen Gel Extraction Kit and subsequently sequenced using the dideoxy-chain-termination method (Sanger sequencing) by Eurofins Genomics (Ebersberg, Germany). Used primers are listed in Table S1.

### 2.3. Quantitative Real-Time RT-PCR (RT-qPCR)

The diagnostic RT-qPCR was carried out using a standard protocol with the QuantiTect Probe RT-PCR Kit (Qiagen) and a primer/probe concentration of 0.8 μM and 0.1 μM, respectively [17]. Primer and probe sequences are depicted in Table S1. The RT was carried out at 50 °C for 30 min. After denaturation/activation step at 95 °C for 15 min, DNA was amplified with 45 cycles at 95 °C (10 s), 55 °C (25 s), and 72 °C (25 s). The assay was performed using the CFX96 Real-Time PCR Detection system according to established protocols [17].

### 2.4. Rapid Amplification of cDNA-Ends (RACE) with PCR

In order to amplify the 3′ and 5′ ends of the viral genome, we slightly modified the 3′- and 5′-RACE Systems (ThermoFisher). The modifications were as follows: SuperScript II reverse transcriptase from the kit was substituted with SuperScript IV reverse transcriptase. PCR amplification was performed using Phusion DNA polymerase. Inosine-containing primers from the kits were replaced by appropriate, inosine-free oligonucleotides. For 5′-RACE, TdT-tailing was first performed using dCTP according to the manufacturer’s instructions. Additionally, a separate tailing reaction was done using dATP. Wherever necessary, DNA was purified using the Qiagen PCR Purification Kit for downstream applications.

### 2.5. Phylogenetic and Sequence Analysis

The novel genome was compared to the updated reference sequences proposed by Smith et al. [7]. Additionally, due to structural similarities, putative HEV-3/HEV-3ra recombinant MG783571 was included in the analysis. For phylogenetic and recombination analyses, the hypervariable region and the rabbit-specific insertion were excluded (between nucleotide positions 2145–2380 and 2834–2835 of MT920909, respectively).

The sequences were aligned using the ClustalW or MUSCLE packages in Geneious Prime v. 2021.0.1 (Bioinformatics Software for Sequence Data Analysis; Biomatters Ltd.; Auckland, New Zealand, 2020). Phylogenetic analysis was conducted in MEGA X (Molecular Evolutionary Genetics Analysis across computing platforms; [18]) using the maximum-likelihood (ML) method [19] with the general time-reversible (GTR) model with gamma distributed rate variation among sites (G) and invariable sites (I) for nucleotide and the Jones–Thornton–Taylor (JTT) model for amino acid sequences with default parameters and 500 bootstrap replicates. Phylogenetic analysis was carried out with complete genome sequences as well as partial sequences as indicated. In the case of amino acid sequences, the maximum-likelihood method and JTT matrix-based model [20] were used. Bootscan recombination analysis [21] was conducted using SimPlot v. 3.5.1 (Stuart C. Ray; Baltimore, MD, USA, 2003).

## 3. Results

### 3.1. Full Genome Sequencing and Determination of Genome Organization

**Figure 1:**
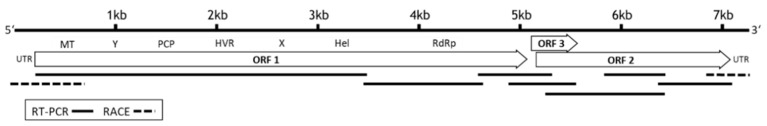
Genome organization and schematic representation of the amplicons used for dideoxy-chain termination (Sanger) sequencing of the complete HEV genome. The genome was determined by sequencing of seven overlapping fragments (solid line), generated by RT-PCR and rapid amplification of cDNA ends (RACE, dotted line). The open reading frame 1 (ORF1) codes for methyltransferase (MT), Y-domain (Y), papain-like cysteine protease (PCP), hypervariable region (HVR), X-domain, RNA helicase (Hel), RNA-dependent RNA polymerase (RdRp), ORF2 codes for capsid protein, and ORF3 for a small accessory protein. Untranslated regions (UTR) are located at the 5′ and 3′ends of the genome.

Seven overlapping fragments were generated from RNA of an HEV positive rabbit (rabbit 081), using a primer set encompassing the whole virus genome (Table S1). These fragments were sequenced and assembled to a complete genome of 7214 nucleotides followed by a poly-A tail (accession number: MT920909). Sequence analysis and alignment with reference strains demonstrated that the genome exhibits the typical features of the *Orthohepevirus A* species group with three open reading frames (ORF1-ORF3), encoding a nonstructural polyprotein (ORF1, nucleotide positions 28–5130), capsid protein (ORF2, nucleotide positions 5165–7150), and the ORF3 protein (nucleotide positions 5154–5498) (Figure 1).

The 5′ UTR consists of 27 nucleotides, but it should be noted that the first three nucleotides (5′-UGG-…3′) might be generated due to terminal transferase activity of the reverse transcriptase in the 5′-RACE reaction [22]. The 3′ UTR consists of 64 nucleotides followed by a poly-A tail.

### 3.2. Sequence and Phylogenetic Analysis

**Figure 2:**
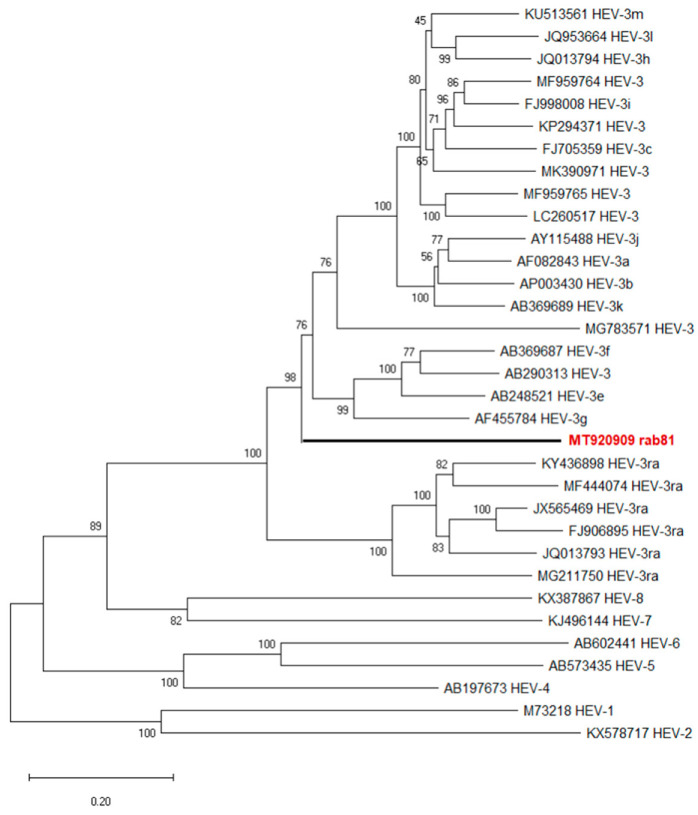
Phylogenetic reconstruction based on complete HEV genomes. Red boldface indicates the novel rabbit HEV strain rab81. The tree represents a maximum-likelihood phylogeny based on the GTR+G+I model with support values at nodes derived from 500 bootstrap repetitions.

Phylogenetic analysis of the novel genome together with the genotype HEV-3 reference sequences according to Smith et al. [7] revealed rab81 at the basal position of the majority of HEV-3 subtypes separate from all original rabbit HEV sequences (HEV-3ra) (Figure 2). Strain MG783571, a putative genotype HEV-3/HEV-3ra recombinant detected in a human plasma sample from France clustered among other HEV-3 subtype genomes.

**Figure 3:**
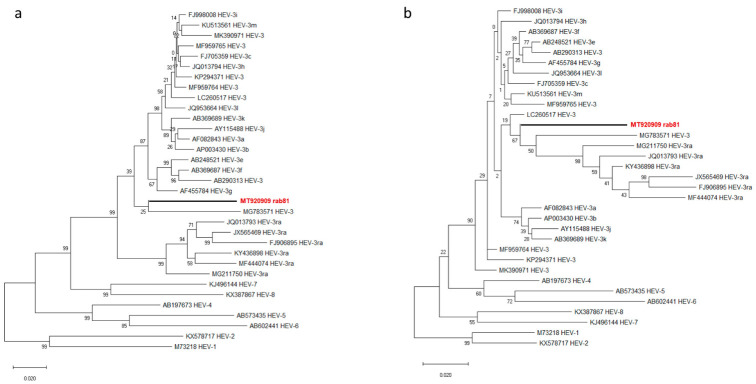
Phylogenetic trees based on amino acid sequences of ORF1-encoded polyprotein ORF1 (**a**) and ORF2-encoded capsid protein (**b**) of HEV. Red boldface indicates the novel rabbit HEV strain. The phylogenies were reconstructed using maximum-likelihood and the Jones–Thornton–Taylor matrix-based model with node support values given from 500 bootstrap replicates.

Further phylogenetic analysis of the amino acid sequences showed ORF1-coded po-lyprotein of rab81 and MG783571 at the basal position of the non-HEV3a HEV-3 subtypes (Figure 3a), while the ORF2-encoded capsid protein sequences of rab81 and MG783571 clustered with all HEV-3ra sequences, but provided very little resolution within this clade (Figure 3b).

The novel genome rab81 showed overall a nucleotide sequence identity of 78.6–80% with HEV-3 subtypes, but only 75% with the reference strain of the HEV-3ra clade (Table 1). The lower similarity with HEV-3ra was consistent for ORF1, ORF2, and ORF3 at the level of the nucleotide sequences as well as on the amino acid sequences of the encoded proteins (Table 1). The HEV recombinant strain MG783571 exhibited amino acid sequence identity of 87.8% (ORF1), 91.8% (ORF2), and 88.6% (ORF3) to the novel rab81 strain (Table 1).

**Figure 4:**
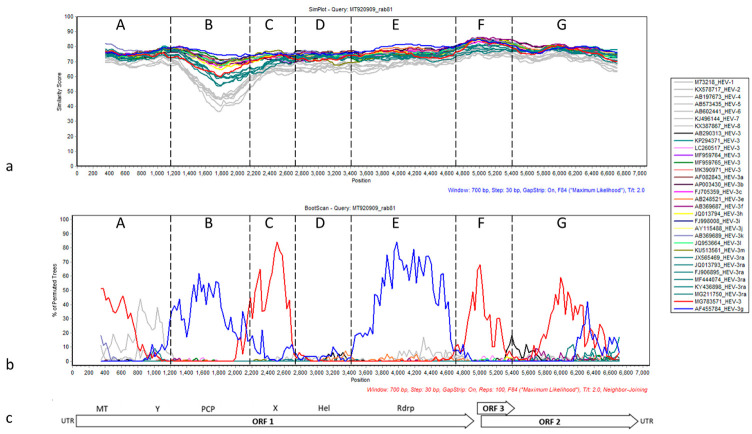
SimPlot analysis of rab81 (MT920909) with similarity plot (**a**) and the result of bootscan analysis (**b**) and a schematic representation of the HEV genome organization as reference (**c**). Note that the hypervariable region was excluded for this analysis. Gray lines refer to HEV genotypes HEV-1, HEV-2, and HEV-4–HEV-8. Teal lines indicate HEV-3ra sequences, blue subgenotype HEV-3g, and red recombinant strain MG783571. The remaining colors were assigned to distinct HEV-3 subgenotypes. The sections A–G were designated based on the peaks of the bootscan plot. See Figure S1 for detailed phylogenetic analyses of the sections.

To determine whether the novel sequence showed evidence of recombination events in its evolutionary history, the HEV-rab81 genome was compared to the reference genomes of all defined HEV-3 subtypes [7] and the potentially recombinant strain MG783571 using Bootscan in SimPlot [21]. The similarity plot showed overall low nucleotide sequence similarity between rab81 and the HEV-3 subgenotype reference sequences along the entire genomes (Figure 4a).

The sequence of rab81 exhibits overall a higher degree of similarity with HEV-3 subtypes than with the remaining genotypes. This is particularly visible between nucleotide positions 1500–2000 (section B) in the similarity plot. This part of the genome contains the relatively diverse protease encoding region and is adjacent to the hypervariable region, which was excluded from the analysis due to a high number of insertions/deletions (indels). In fact, this region shows the highest similarity for non-rabbit HEV-3 subtypes, followed by HEV-3ra, and finally by the remaining HEV genotypes. A similar pattern can be observed in the highly conserved ORF2/ORF3 overlap region at about nucleotide position 5000 (section F), where the similarity to non-rabbit HEV-3 subtypes is higher compared to all other sequences. Bootscan analysis indicated a mosaic-like genome composition with six putative recombination breakpoints and alternating sections of rab81 clustering with HEV-3g (AF455784, blue line) and recombinant HEV-3 (MG783571, red line) sequences (Figure 4b). These sections are roughly equivalent to the conserved domains within HEV ORF1-encoded protein described by Koonin et al. [23]. Phylogenetic reconstructions based on the sequence sections between putative recombination breakpoints supported the closer local similarity of rab81 with the mentioned sequences although node support was generally low (Figure S1a–g).

**Figure 5:**
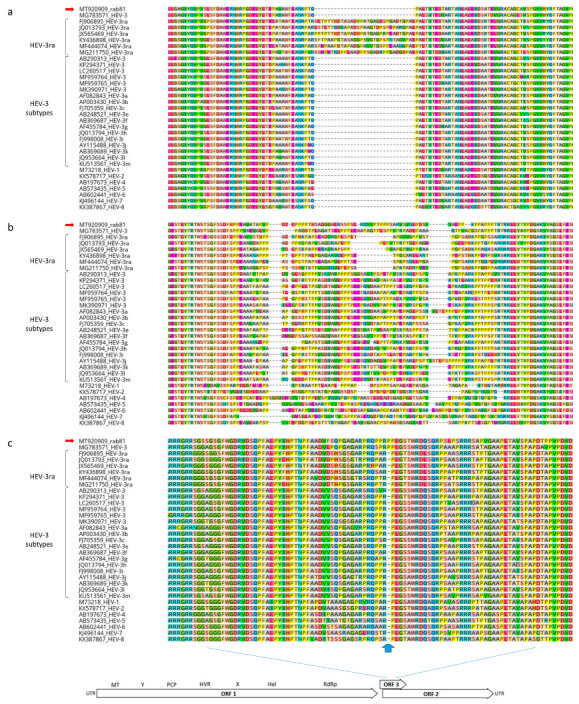
Amino acid sequence alignments of X-region (**a**) and hypervariable region of ORF1-encoded polyprotein (**b**), and capsid protein encoded by ORF2 within the ORF2/ORF3 overlap region (**c**). The X-region of ORF1 refers to nucleotide positions 2776–2932 of MT920909 (strain rab81). The hypervariable region is flanked by conserved TRTWS and RRLL amino acid sequences (refers to nucleotide positions 2146–2379 of MT920909, strain rab81). The position of the proline insertion within the ORF2-encoded capsid protein amino acid sequence refers to nucleotide positions 5408–5410.

The sequence of rab81 exhibited additional notable properties that differ from the typical pattern in HEV-3ra strains: First, the absence of the 90 nt insertion within the X-region of ORF1 (nucleotide positions 2776–2932), which is characteristic for other rabbit-derived HEV sequences (Figure 5a). This insertion is also absent in recombinant strain MG783571 and otherwise only absent in non-rabbit HEV-3 strains. In addition, the hypervariable region (HVR) which is flanked by conserved N-terminal TSGFSS and C-terminal RRLL amino acid sequences (corresponding to nucleotide positions 2155–2388), contains a unique 82 amino acid stretch which does not match with any of the HEV-3ra HVR domains (Figure 5b). Finally, the capsid protein-encoding ORF2 sequence contains within the ORF2/ORF3 overlapping regions a unique proline codon insertion (nucleotide positions 5412–5414), which is also found in strain MG783571 (Figure 5c).

Finally, a unique nucleotide exchange (G/A, position 5311) is found in a highly conserved ORF2/ORF3 overlapping region, which is the target region for widely used real-time RT-PCR [17,24]. Modification of the original probe sequence (5′-TGATTCTCAGCC CTT CGC-3′, [17]) to the rab81 adapted probe sequence (5′-TGATTCTC AACCCTTCGC-3′) leads to lowered threshold cycle (ct) values (on average 3 ct values) and thus substantially increased sensitivity as demonstrated for a plasmid template encoding the rab81 structure protein region (Figure S2, Table S1).

## 4. Discussion

We determined the complete genome sequence of an HEV strain detected in the liver of a rabbit [12]. Initial phylogenetic analysis based on a short partial sequence of the RdRp coding region assigned the strain to subtype HEV-3g [12]. However, analysis of the entire genome sequence demonstrated a generally low sequence similarity to previously described HEV-3 subtypes. In particular, it does not cluster within the HEV-3ra clade, but at the base of a phylogenetic branch with the remaining HEV-3 subtypes and constitutes a putative new subtype or recombinant strain.

The novel rab81 strain displays characteristics that are similar to that of a recently described genotype HEV-3 recombinant strain (MG783571), which was detected in a human plasma sample from France [25]. This strain also clusters phylogenetically outside HEV-3ra clade and at the base or among the remaining HEV-3 subtypes. The nucleotide sequence identity of the complete genomes of both strains is about 77.8%. Both strains harbor a unique proline codon insertion within ORF2 in the ORF2/ORF3 overlapping region, both lack the HEV-3ra typical 90 nucleotide insertion and exhibit a unique insertion within the HVR region. Further, MG783571 is proposed to carry a mosaic genome derived from rabbit HEV-3ra and other HEV-3 subgenotype sequences [25], and different parts of the genome showed different affinities in our section-wise phylogenies (Figure S1). Similarly, in our analysis, the comparison of the rab81 sequence with the HEV-3 subgenotype reference sequences revealed several possible recombination events. However, the bootstrap support values in these analyses are too low to decide conclusively whether the rab81 strain is a recombinant. Therefore, we conclude that rab81 may represent a novel subgenotype. Since the rab81 sequence contains a single nucleotide exchange in the probe binding region of commonly used RT-qPCR assays [17,24], a probe sequence should be adapted or alternatively broad-spectrum RT-PCR protocols should be applied [17,26].

Finally, these findings further stress the role of rabbits as sources for novel zoonotic HEV strains. The transmissibility of rabbit HEV strains has been already confirmed experimentally, where HEV strains isolated from rabbits were successfully inoculated to pigs [27] as well as cynomolgus macaques [28] with subsequent productive replication. Furthermore, in France and Switzerland, immunosuppressed patients who suffered from persistent infections were infected by HEV-3ra strains [14,16,29]. Since none of the patients was exposed directly to rabbits, the presumed origin of the infection seemed to be a foodborne infection probably due to consumption of meat and liver of infected animals or derived products. However, the detection of HEV-3ra infected rabbits in urban regions also shows that there is potential for indirect transmissions [30].

## 5. Conclusions

In summary the results, obtained from phylogenetic, SimPlot, bootscan, and amino acid sequence analyses revealed a putative novel rabbit-derived HEV-3 subgenotype. Further studies will have to evaluate the virulence and infectivity of the novel strain by using cell culture systems and inoculation in animal models.

## Figures and Tables

**Table 1:** Nucleotide (nt) and amino acid (aa) sequence identities (in %) between novel strain rab81 (MT920909), reference HEV strains within genotype HEV-3, and the putative recombinant strain MG783571.

HEV-3 Subtype	Accession Number	Complete Genome	ORF1		ORF2		ORF3	
		nt	nt	aa	nt	aa	nt	aa
HEV-3a	AF082843	78.6	77.4	89.8	81.6	93.0	90.4	87.7
HEV-3b	AP003430	78.6	77.5	90.3	81.6	92.9	90.6	89.5
HEV-3c	FJ705359	78.7	77.6	90.0	81.6	93.2	88.9	84.2
HEV-3e	AB248521	79.3	78.1	90.2	82.1	93.5	88.9	86.8
HEV-3f	AB369687	79.8	78.2	90.2	82.3	93.5	90.1	89.5
HEV-3g	AF455784	80.0	79.2	90.7	82.3	92.9	89.2	88.6
HEV-3h	JQ013794	78.6	77.3	90.4	81.9	93.2	91.5	89.5
HEV-3i	FJ998008	79.0	77.9	89.8	81.9	93.6	90.4	90.4
HEV-3j	AY115488	78.8	77.4	89.2	82.1	92.9	90.1	88.6
HEV-3k	AB369689	79.8	78.9	90.7	81.9	92.9	90.7	90.4
HEV-3l	JQ953664	79.2	77.9	90.2	82.0	93.2	90.4	90.4
HEV-3m	KU513561	78.8	77.3	89.4	82.1	94.3	91.2	88.6
HEV-3ra	FJ906895	75.3	73.4	84.8	79.8	89.7	83.8	80.7
HEV-3	AB290313	79.4	78.1	89.3	82.6	92.7	88.1	86.8
HEV-3	KP294371	79.4	78.2	89.6	82.4	92.4	87.8	86.8
HEV-3	LC260517	79.0	77.6	89.9	82.2	94.0	89.7	83.4
HEV-3	MF959764	79.1	78.1	89.9	81.7	93.5	89.3	86.0
HEV-3	MF959765	79.2	78.0	89.8	82.2	92.7	89.6	87.7
HEV-3	MK390971	79.1	77.7	89.8	82.6	92.6	89.3	86.8
HEV-3	MG783571	77.8	76.2	87.8	81.2	91.8	91.2	88.6

## Data Availability

All data of this study are available within this manuscript and its Supplementary Material.

## Supplementary Materials

The following are available online at https://www.mdpi.com/article/10.3390/v13061065/s1, Table S1: Primers used for HEV RNA quantification, genotyping, and genome sequencing, Figure S1: Molecular phylogenetic analysis of seven partial HEV sequences (a–g) as determined by SimPlot analysis, Figure S2: Nucleotide sequence alignment of quantitative real-time RT-PCR (RT-qPCR) probe target region (a), RT-qPCR runs (triplicate) at three dilution steps (b), and mean ct values yielded by original probe or adapted probe (c).
