# Supplementary material for: A Putative Novel Hepatitis E Virus Genotype 3 Subtype Identified in Rabbit, Germany 2016
Source: Viruses. 2021 Jun 3;13(6):1065. doi: 10.3390/v13061065 (PMC8228152; doi:10.3390/v13061065)
Supplement: Supplementary file 1 [file viruses-13-01065-s001.zip › viruses-1232741-supplementary.pdf]

Table S1 : Primers used for HEV RNA quantification, genotyping, and genome sequencing

Name	Sequence	nucleotide position*	use	Reference
JVHEVR	AGGGGTTGGTTGGATGAA	5348 - 5331	qPCR	Jothikumar et al., 2006
JVHEVF	GGTGGTTTCTGGGGTGAC	5279 - 5926	qPCR	Jothikumar et al., 2006
JVHEV.P81	TGATTCTCAACCCTTCGC	5302 - 5319	qPCR	this work
JVHEV.P	TGATTCTCAGCCCTTCGC	5302 - 5319	qPCR	Jothikumar et al., 2006
HEV.R	GCGAAGGGGTTGGTTGGATG	5325 - 5333	RT-PCR/Sequencing	Vina-Rodriguez et al., 2015
HEV.RdRp_F2b	GTGCTCTGTTTGGCCCNTGGTTYMG	4280 - 4304	RT-PCR/Sequencing	Hammerschmidt et al., 2017
R5674	TGAGTGTTGGTGCCGTCCTG	5685 - 5666	Sequencing	this work
R5625	CATACAAGACAAGATTAGTGCC	5636 - 5615	RT-PCR/Sequencing	this work
F5086	GGACCTTACAAATTCTATTATACAG	5094 - 5118	Sequencing	this work
F5048	GGCAAGGCTCACTTTACCGA	5056 - 5075	RT-PCR/Sequencing	this work
AAP mod	GGCCACGCGTCGACTAGTACGGGGGGGGGGGGGGGG	RACE adapter	Sequencing	ThermoFisher (adapted)
AP	GGCCACGCGTCGACTAGTACTTTTTTTTTTTTTTTTT	RACE adapter	Sequencing	ThermoFisher
AUAP	GGCCACGCGTCGACTAGTAC	RACE adapter	Sequencing	ThermoFisher
R6591	GAAAATGGTCTGATCGTACTC	6622 - 6602	RT-PCR/Sequencing	this work
R6578	GAGAGCCACAACACATCATT	6588 - 6569	Sequencing	this work
R5926	CCTGGTTACGATAATGGAGG	5936 - 5917	Sequencing	this work
R2429	CACAATCCGACTCGAATAAGG	2449 - 2429	Sequencing	this work
K081.RACE5'700	GACAACTACGCGACTTCCATC	684 - 664	Sequencing	this work
K081.RACE5'400	CGCGAGTTGGGGCAGAGTAC	400 - 381	Sequencing	this work
K081.RACE3'300	GGCCCTGTGTCCATTTCTGC	6938 - 6957	Sequencing	this work
F5906	CCTCCATTATCGTAACCAGG	5917 - 5936	RT-PCR/Sequencing	this work
F1610	CTTGAGGCCCTTTATAGTGC	1609 - 1628	Sequencing	this work
R6506	GTCCTGTTCATGTTGATTGTCG	6517 - 6496	RT-PCR/Sequencing	this work
R3157	CAGCAAATGCGGTGGTAATGAC	3165 - 3144	Sequencing	this work
F6485	CGACAATCAACATGAACAGGAC	6496 - 6517	RT-PCR/Sequencing	this work
F5305	CCTTCGCCCTCCCCTATATTC	5313 - 5333	RT-PCR/Sequencing	this work
F960	CATTCCACGCAGTCCCAGTTC	959 - 979	Sequencing	this work
R1042	CGAGCAACAAAAGGCCTGATC	1041 - 1021	Sequencing	this work
R725	CATCATGGTTATACCCTGCACTAG	724 - 701	Sequencing	this work
F2991	CTATCCAGCAGGGGGATGTTG	2999 - 3019	RT-PCR/Sequencing	this work
F705	GTGCAGGGTATAACCATGATG	704 - 724	Sequencing	this work
F32	GAGGCCCATCAGTTTATTAAGG	31 - 52	RT-PCR/Sequencing	this work
terR	CTCCCGGGTYTTRCCTACCYTC	7144 - 7123	RT-PCR/Sequencing	this work
4R	CACATAAAATGTTTTAGAATGC	6781 - 6760	Sequencing	this work
3R	CATAAGACCATTGAATCATCTC	4718 - 4697	RT-PCR/Sequencing	this work
3F	GAGATGATTCAATGGTCTTATG	4697 - 4718	Sequencing	this work
2R	GCCGTAGCAATAATAGTAGTC	3518 - 3498	RT-PCR/Sequencing	this work
2F	GACTACTATTATTGCTACGGC	3498 -3518	Sequencing	this work

***numbering according to MT920909**

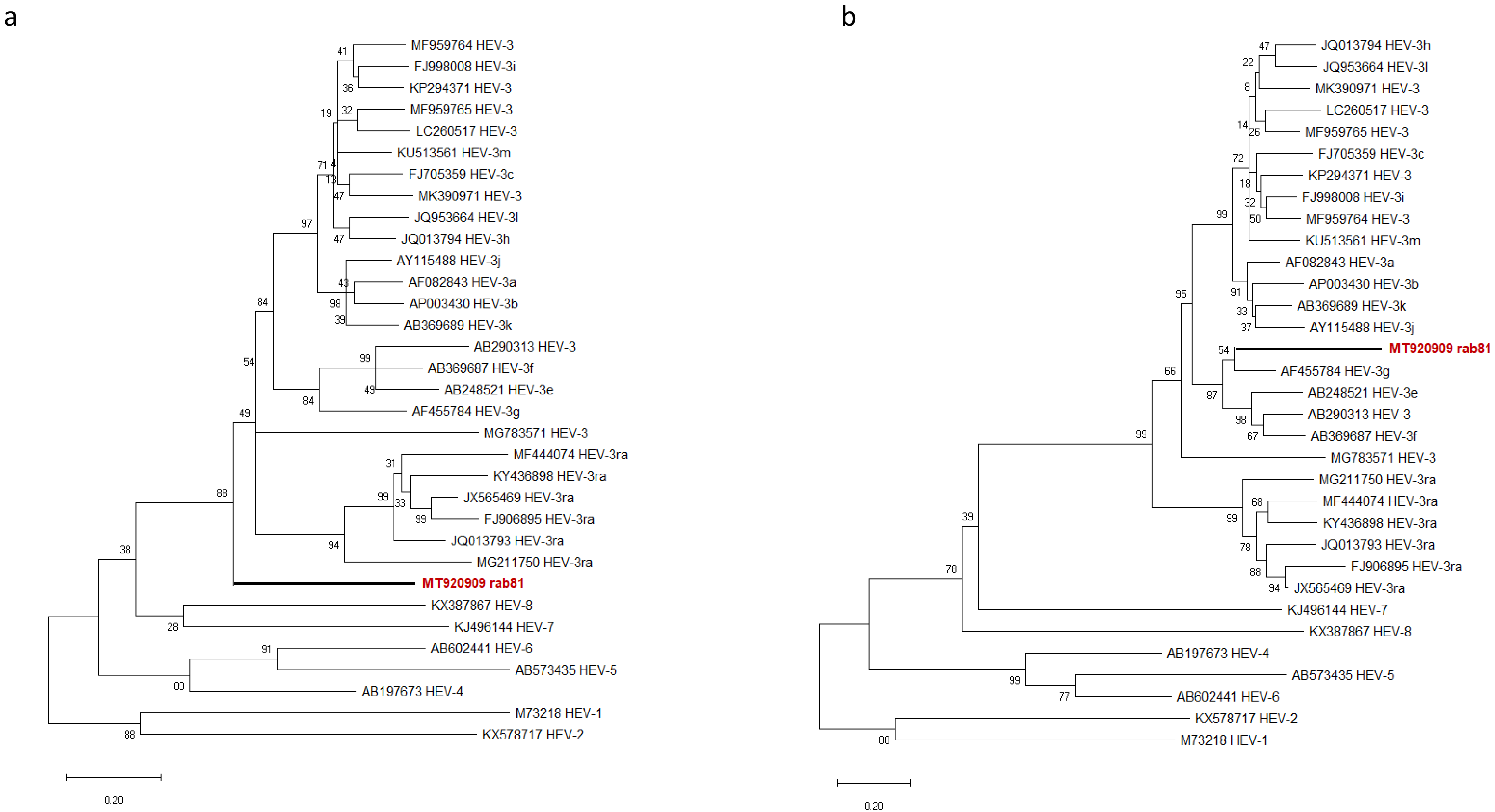

Figure S1: Molecular phylogenetic analysis of seven partial HEV sequences (a-g) as determined by SimPlot analysis

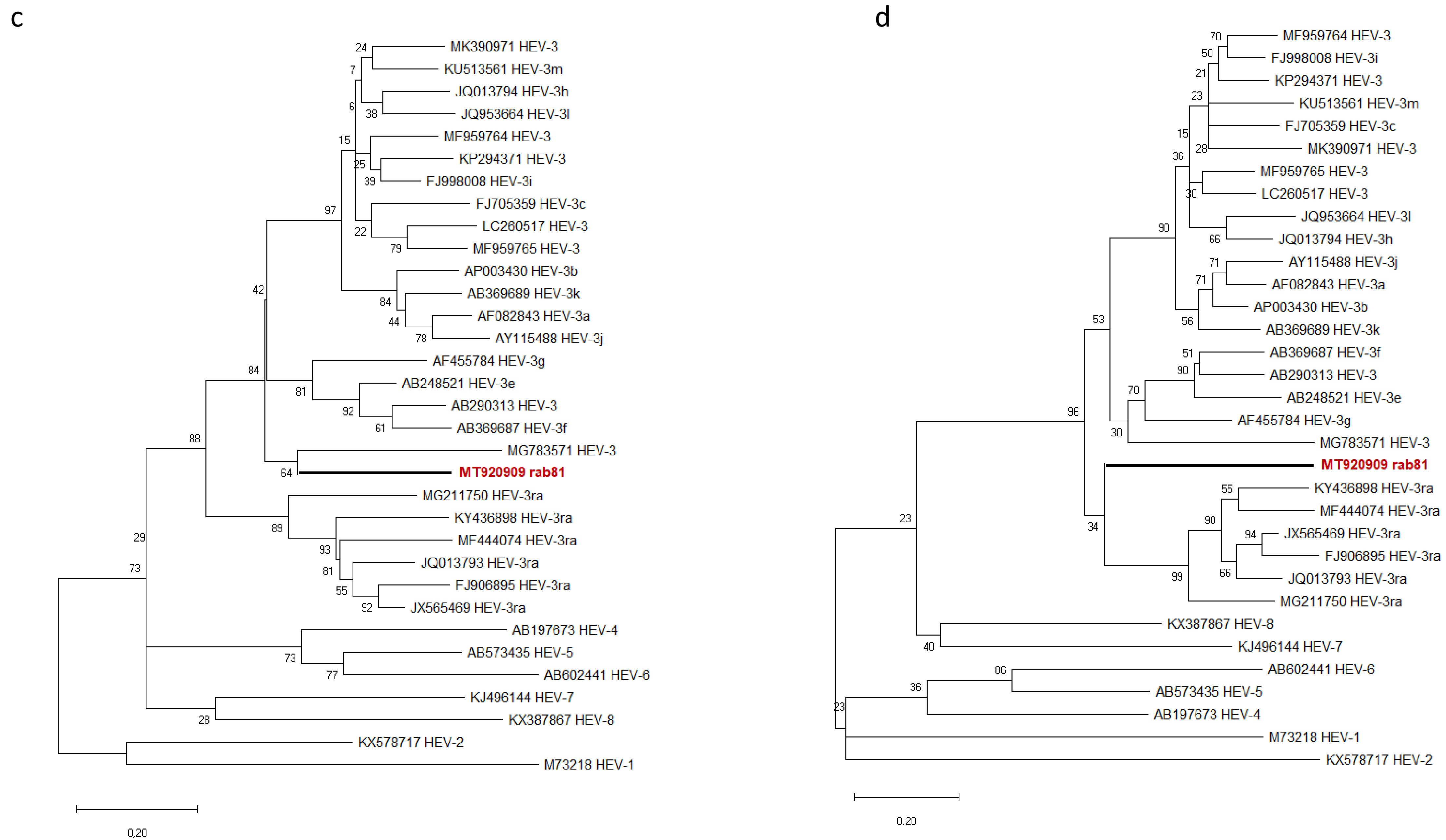
c
MK390971 HEV-3
KU513561 HEV-3m
JQ013794 HEV-3h
JQ953664 HEV-3l
MF959764 HEV-3
KP294371 HEV-3
FJ998008 HEV-3i
FJ705359 HEV-3c
LC260517 HEV-3
MF959765 HEV-3
AP003430 HEV-3b
AB369689 HEV-3k
AF082843 HEV-3a
AY115488 HEV-3j
AF455784 HEV-3g
AB248521 HEV-3e
AB290313 HEV-3
AB369687 HEV-3f
MG783571 HEV-3
MT920909 rab81
MG211750 HEV-3ra
KY436898 HEV-3ra
MF444074 HEV-3ra
JQ013793 HEV-3ra
FJ906895 HEV-3ra
JX565469 HEV-3ra
AB197673 HEV-4
AB573435 HEV-5
AB602441 HEV-6
KJ496144 HEV-7
KX387867 HEV-8
KX578717 HEV-2
M73218 HEV-1
0,20
d
MF959764 HEV-3
FJ998008 HEV-3i
KP294371 HEV-3
KU513561 HEV-3m
FJ705359 HEV-3c
MK390971 HEV-3
MF959765 HEV-3
LC260517 HEV-3
JQ953664 HEV-3l
JQ013794 HEV-3h
AY115488 HEV-3j
AF082843 HEV-3a
AP003430 HEV-3b
AB369689 HEV-3k
AB369687 HEV-3f
AB290313 HEV-3
AB248521 HEV-3e
AF455784 HEV-3g
MG783571 HEV-3
MT920909 rab81
KY436898 HEV-3ra
MF444074 HEV-3ra
JX565469 HEV-3ra
FJ906895 HEV-3ra
JQ013793 HEV-3ra
MG211750 HEV-3ra
KX387867 HEV-8
KJ496144 HEV-7
AB602441 HEV-6
AB573435 HEV-5
AB197673 HEV-4
M73218 HEV-1
KX578717 HEV-2
0.20

S1

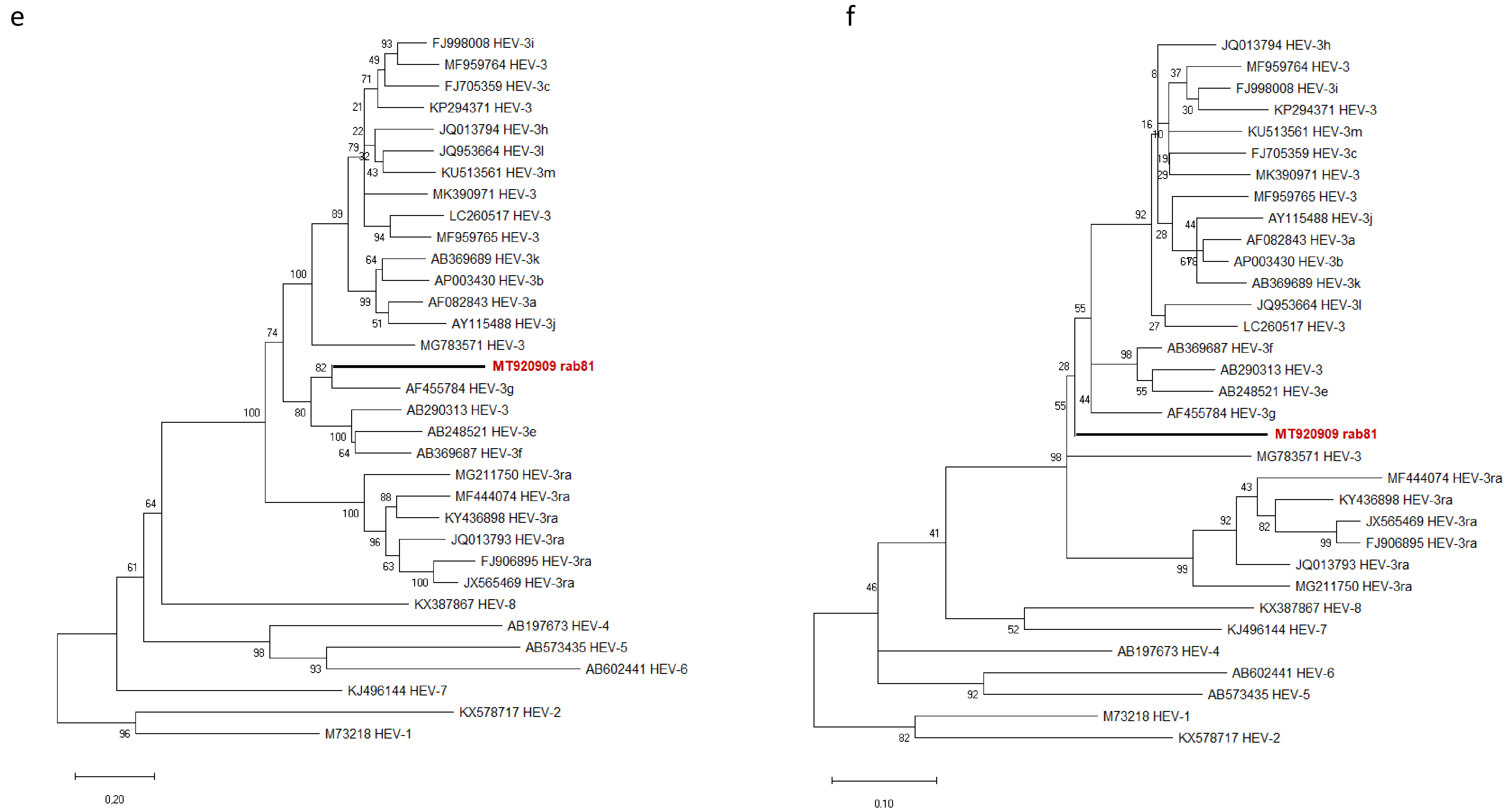

e
FJ998008 HEV-3i
MF959764 HEV-3
FJ705359 HEV-3c
KP294371 HEV-3
JQ013794 HEV-3h
JQ953664 HEV-3l
KU513561 HEV-3m
MK390971 HEV-3
LC260517 HEV-3
MF959765 HEV-3
AB369689 HEV-3k
AP003430 HEV-3b
AF082843 HEV-3a
AY115488 HEV-3j
MG783571 HEV-3
MT920909 rab81
AF455784 HEV-3g
AB290313 HEV-3
AB248521 HEV-3e
AB369687 HEV-3f
MG211750 HEV-3ra
MF444074 HEV-3ra
KY436898 HEV-3ra
JQ013793 HEV-3ra
FJ906895 HEV-3ra
JX565469 HEV-3ra
KX387867 HEV-8
AB197673 HEV-4
AB573435 HEV-5
AB602441 HEV-6
KJ496144 HEV-7
KX578717 HEV-2
M73218 HEV-1
0.20
f
JQ013794 HEV-3h
MF959764 HEV-3
FJ998008 HEV-3i
KP294371 HEV-3
KU513561 HEV-3m
FJ705359 HEV-3c
MK390971 HEV-3
MF959765 HEV-3
AY115488 HEV-3j
AF082843 HEV-3a
AP003430 HEV-3b
AB369689 HEV-3k
JQ953664 HEV-3l
LC260517 HEV-3
AB369687 HEV-3f
AB290313 HEV-3
AB248521 HEV-3e
AF455784 HEV-3g
MT920909 rab81
MG783571 HEV-3
MF444074 HEV-3ra
KY436898 HEV-3ra
JX565469 HEV-3ra
FJ906895 HEV-3ra
JQ013793 HEV-3ra
MG211750 HEV-3ra
KX387867 HEV-8
KJ496144 HEV-7
AB197673 HEV-4
AB602441 HEV-6
AB573435 HEV-5
M73218 HEV-1
KX578717 HEV-2
0.10

S1

g

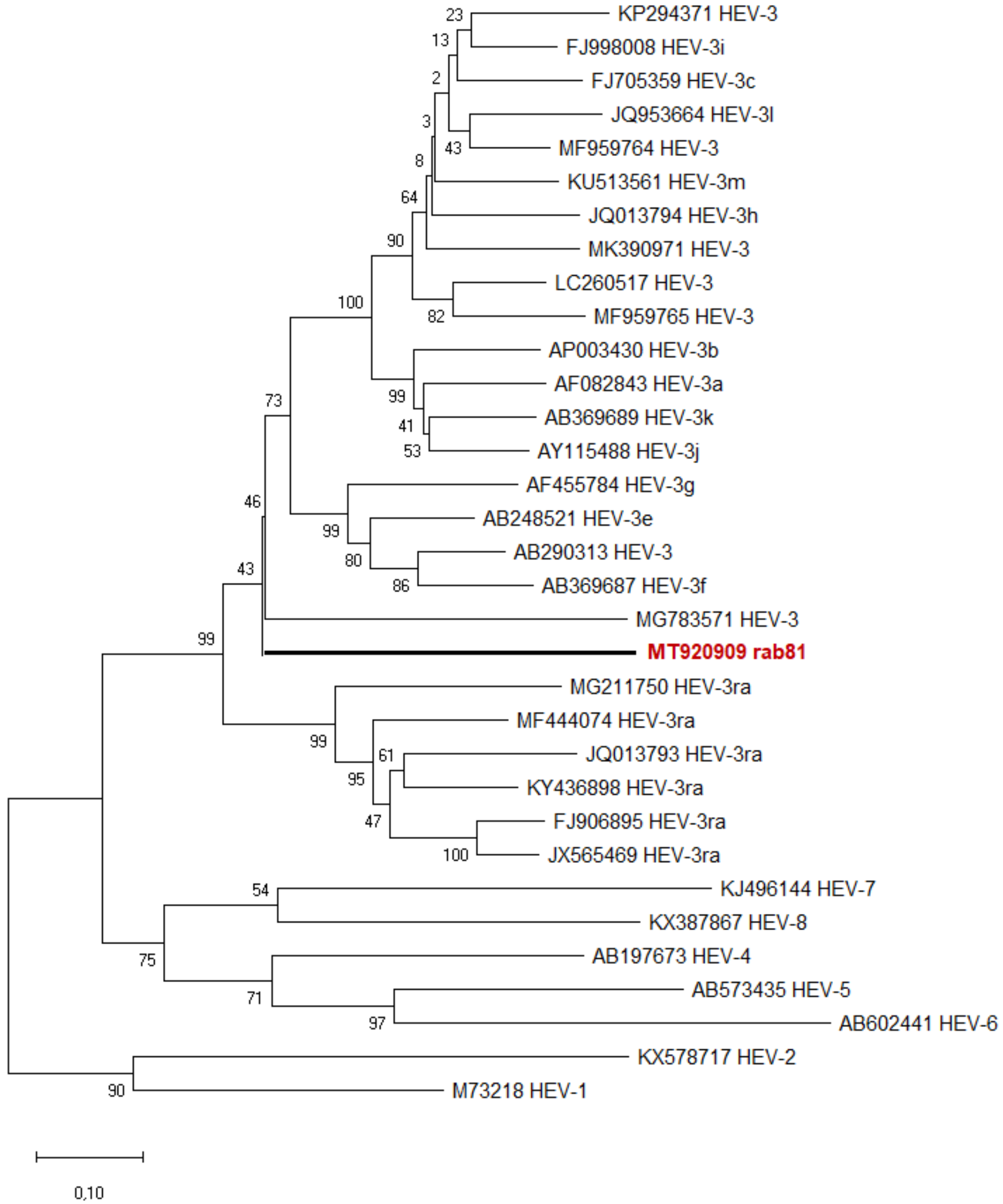
KP294371 HEV-3
FJ998008 HEV-3i
FJ705359 HEV-3c
JQ953664 HEV-3l
MF959764 HEV-3
KU513561 HEV-3m
JQ013794 HEV-3h
MK390971 HEV-3
LC260517 HEV-3
MF959765 HEV-3
AP003430 HEV-3b
AF082843 HEV-3a
AB369689 HEV-3k
AY115488 HEV-3j
AF455784 HEV-3g
AB248521 HEV-3e
AB290313 HEV-3
AB369687 HEV-3f
MG783571 HEV-3
MT920909 rab81
MG211750 HEV-3ra
MF444074 HEV-3ra
JQ013793 HEV-3ra
KY436898 HEV-3ra
FJ906895 HEV-3ra
JX565469 HEV-3ra
KJ496144 HEV-7
KX387867 HEV-8
AB197673 HEV-4
AB573435 HEV-5
AB602441 HEV-6
KX578717 HEV-2
M73218 HEV-1
0,10

S1

a

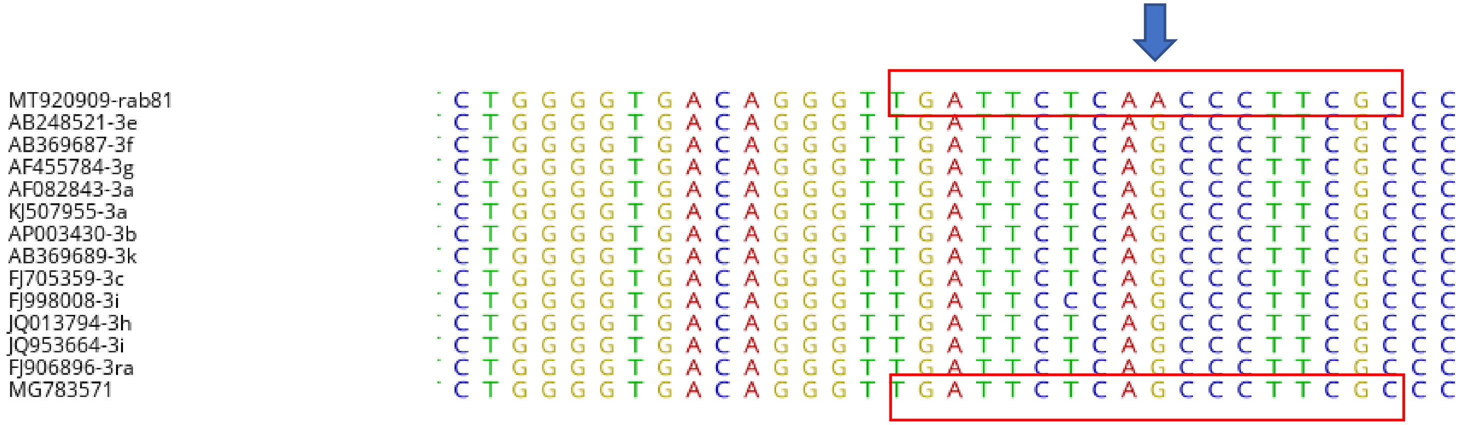

b

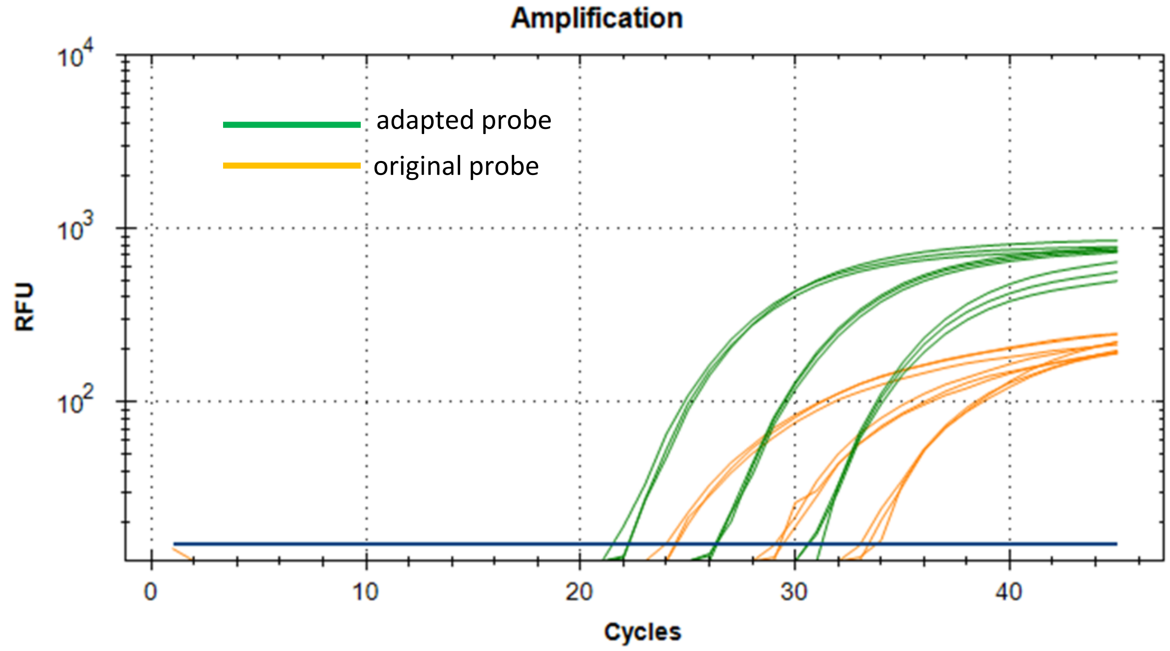

c

	ct value (average of three)	
dilution	adapted probe	original probe
10^{-3}	30.9	33.4
10^{-4}	26.3	29.2
10^{-5}	22.0	24.3

Figure S2: Nucleotide sequence alignment of quantitative real-time RT-PCR (qRT-PCR) probe target region (a), qRT-PCR runs (triplicate) at three dilution steps (b) and bean ct-values yielded by original probe or adapted probe (c)
